# Neandertal introgression partitions the genetic landscape of neuropsychiatric disorders and associated behavioral phenotypes

**DOI:** 10.1038/s41398-022-02196-2

**Published:** 2022-10-05

**Authors:** Michael Dannemann, Yuri Milaneschi, Danat Yermakovich, Victoria Stiglbauer, Hanna Maria Kariis, Kristi Krebs, Manuel A. Friese, Christian Otte, Estonian Biobank Research Team, Tõnu Esko, Andres Metspalu, Lili Milani, Reedik Mägi, Mari Nelis, Kelli Lehto, Brenda W. J. H. Penninx, Janet Kelso, Stefan M. Gold

**Affiliations:** 1 Institute of Genomics, University of Tartu, Tartu, Estonia; 2 Max Planck Institute for Evolutionary Anthropology, Leipzig, Germany; 3 Department of Psychiatry, Amsterdam Public Health and Amsterdam Neuroscience, Amsterdam UMC/Vrije Universiteit, Amsterdam, The Netherlands; 4 Charité – Universitätsmedizin Berlin, Freie Universität Berlin, Humboldt-Universität zu Berlin, and Berlin Institute of Health, Klinik für Psychiatrie und Psychotherapie, Campus Benjamin Franklin, Berlin, Germany; 5 Institut für Neuroimmunologie und Multiple Sklerose (INIMS), Zentrum für Molekulare Neurobiologie, Universitätsklinikum Hamburg Eppendorf, Hamburg, Germany; 6 Charité – Universitätsmedizin Berlin, Freie Universität Berlin, Humboldt-Universität zu Berlin, and Berlin Institute of Health, Medizinische Klinik m.S. Psychosomatik, Campus Benjamin Franklin, Berlin, Germany

**Keywords:** Genetics, Clinical genetics

## Abstract

Despite advances in identifying the genetic basis of psychiatric and neurological disorders, fundamental questions about their evolutionary origins remain elusive. Here, introgressed variants from archaic humans such as Neandertals can serve as an intriguing research paradigm. We compared the number of associations for Neandertal variants to the number of associations of frequency-matched non-archaic variants with regard to human CNS disorders (neurological and psychiatric), nervous system drug prescriptions (as a proxy for disease), and related, non-disease phenotypes in the UK biobank (UKBB). While no enrichment for Neandertal genetic variants were observed in the UKBB for psychiatric or neurological disease categories, we found significant associations with certain behavioral phenotypes including pain, chronotype/sleep, smoking and alcohol consumption. In some instances, the enrichment signal was driven by Neandertal variants that represented the strongest association genome-wide. SNPs within a Neandertal haplotype that was associated with smoking in the UKBB could be replicated in four independent genomics datasets.

Our data suggest that evolutionary processes in recent human evolution like admixture with Neandertals significantly contribute to behavioral phenotypes but not psychiatric and neurological diseases. These findings help to link genetic variants in a population to putative past beneficial effects, which likely only indirectly contribute to pathology in modern day humans

## Introduction

It has long been known that psychiatric disorders run in families, indicating substantial heredity, but their genetic basis has remained elusive for decades [1]. This has changed only fairly recently with the advent of large consortia that have discovered and successfully replicated genome-wide associations of common gene variants (single nucleotide polymorphisms, SNPs) for several disorders including schizophrenia [2] and major depression [3–6].

**Fig. 1:**
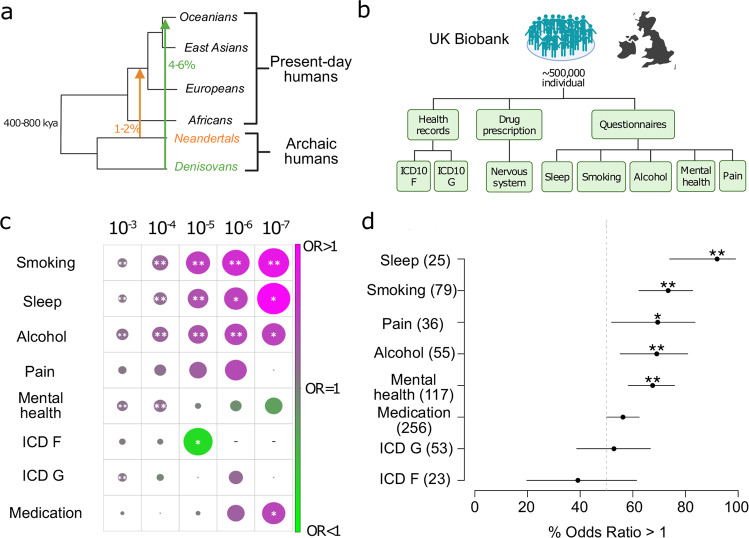
The proportional association of Neandertal DNA with mental and behavioral disease and non-disease phenotypes. **a** Some of the genetic variation in present-day people is a result of the admixture with archaic humans such as Neandertals and Denisovans ~55,000 years ago. Consequently, ~2% of the genomes of present-day non-Africans are of Neandertal ancestry and an additional 5% of the genomes of Oceanians derived from Denisovans. Approximately 40% of the genome of Neandertals can be reconstructed in people today and tested for its phenotypic potential in genome-wide association studies. **b** Overview of UK Biobank GWAS summary statistics for behavioral phenotypes that were included in this study. Included phenotypic information was derived from medical records that include diagnosis and drug prescription information and questionnaires related to behavioral patterns. **c** The combined numbers of associations of tag aSNPs with all phenotypes from one of eight tested groups in the UK Biobank (*y*-axis) was put in relation to the average of the combined numbers of associations for 1,000 sets of frequency-matched non-archaic tag SNPs. The resulting average odds ratios based on five significance cutoffs (shown on top) are displayed and color-coded and shown in size proportional to the corresponding log of the average odds ratio. Instances where tag aSNPs showed significant deviations from the number of phenotype associations of their non-archaic counterparts were highlighted (*P < 0.05, **FDR < 0.05). **d** The average odds ratio between the number of associations of tag aSNPs compared to the number of associations for 1,000 sets of frequency-matched non-archaic tag SNPs for each individual phenotype from eight phenotype groups from the UK Biobank were calculated. The proportion of these odds ratios that are larger than one for a given phenotype group *(y*-axis, number of tests across all significance cutoffs for each group shown in parenthesis), together with the binomial 95% confidence intervals are displayed. Phenotype groups for which this proportion is significantly different from the random expectation of 50% are highlighted (*P < 0.05, **FDR < 0.05).

While evolutionary origins for the susceptibility to psychiatric [7] and neurological [8] diseases have been postulated, this hypothesis has not been tested directly. One approach to address this question is to uncover the evolutionary history of phenotype-associated variation. One process through which this variation could have entered a population is through admixture events at some point in the past. Here, introgressed variants from archaic humans can serve as an intriguing research paradigm. After modern humans left Africa more than 60,000 years ago, genetic evidence suggests multiple admixture events ~55,000 years [9] between modern humans and now extinct archaic humans including Neandertals [10–12] and Denisovans [13, 14]. After admixture, early events of negative selection removed large parts of archaic DNA but ~2% of Neandertal ancestry is still found in all present-day non-Africans [15–17]. Some of the remaining Neandertal variants have reached high frequencies in some present-day populations, suggesting that they might have conferred advantages at some point since admixture [12, 18, 19]. Introgressed Neandertal variants are also particularly interesting because they are detectable in all non-African populations [14, 20] and their phenotypic correlates can thus be studied across different ancestries (e.g. European, Asian), see Fig. 1a.

In order to identify complex traits that have been significantly influenced by Neandertal DNA, previous studies have compared their numbers of associations of introgressed archaic variants in GWAS to the associations of frequency-matched non-archaic variants. In one study that analyzed health record data in ~28,000 individuals, the authors demonstrated that neurological and psychiatric disorders showed the highest proportional Neandertal DNA contribution [21]. In addition, a second study has shown that among 136 diverse non-disease phenotypes, tested in ~122,000 individuals of the UK Biobank pilot release, Neandertal DNA was over-proportionally associated with two mood-related traits, sleeping patterns and smoking status [22]. It has been postulated that due to the clinical heterogeneity of complex human disorders, and psychiatric syndromes in particular, careful behavioral phenotyping might yield more robust biological substrates [23]. However, a direct comparison of how archaic DNA contributes to diagnostic entities vs. related but non-disease phenotypes is lacking.

To address this question, we conducted a series of analyses of GWAS summary statistics analyses in the UK Biobank (UKBB) [24], comparing associations of Neandertal variants with human CNS disorders, drug prescriptions as a proxy for disease, and non-disease phenotypes (see Fig. 1b). We also leveraged data from two additional cohorts with sufficient genetic coverage of Neandertal variants and available deep phenotyping, the Netherlands Study of Depression and Anxiety (NESDA) [25] and the Biobank Japan [26]. Finally, we identified several Neandertal risk factors that strongly influence these traits and demonstrate that some of them are population-specific.

## Methods

### UK Biobank

Summary statistics for 261 genome-wide association studies (GWAS, Table S1) from the UK Biobank [24] were obtained from the Neale lab [http://www.nealelab.is/uk-biobank/]. A detailed description of the analyses underlying these GWAS statistics can be found at http://www.nealelab.is/blog/2017/9/11/details-and-considerations-of-the-uk-biobank-gwas and http://www.nealelab.is/blog/2019/10/24/updating-snp-heritability-results-from-4236-phenotypes-in-uk-biobank.

GWAS have been conducted using 361,194 biobank individuals genotyped at ∼10.8 million SNPs using custom arrays and imputation based on the Haplotype Reference Consortium, the 1000 Genomes Project, and UK10K, and that have passed quality control filters (see Supplementary Methods for a more detailed description).

These results are publicly available with no restrictions on their use.

### Biobank Japan

We used publicly available summary statistics for the four smoking GWAS from the Biobank Japan [27], that matched phenotypes we analyzed in the UK Biobank. These results are publicly available with no restrictions on their use.

The smoking phenotypes have been defined as (1) ever versus never smokers, (2) smoking cessation, (3) age of smoking initiation and (4) quantity of smoking. These GWAS have been conducted in ~200,000 individuals from the Biobank Japan cohort, with up to 165,436 individuals per GWAS. Individuals were genotyped using custom arrays and imputation based on the 1000 Genomes, and included 5,826,586 SNPs after application of quality filters (see Supplementary Methods for a more detailed description).

### The Netherlands Study of Depression and Anxiety (NESDA)

We generated GWAS summary statistics for eight behavioral phenotypes (Table S1). These analyses were conducted in subsets of individuals of the NESDA cohort, ranging between 1842 and 2261 individuals. Genotype data was generated based on custom genotyping arrays and subsequent imputation and included 8,657,974 SNPs after quality filtering. Genome-wide association analyses assuming an additive model were carried out using SNPTEST [28]. More detailed information on the GWAS can be found in the Supplementary Methods section. The NESDA research protocol was approved by the ethical committee of participating universities. The current analyses are covered by the informed consent of the participants and carried out under the approved protocol NESDA DAP19-47 (1947_Dannemann_Gold).

### Estonian Biobank

We conducted GWAS to obtain the summary statistics for smoking history from participants of the Estonian Biobank cohort [29]. The phenotype was defined as ever versus never smokers. Samples were genotyped using the Illumina Global Screening Arrays and imputation was based on the Estonian population-specific imputation reference (see Supplementary Methods for a more detailed description). These analyses were covered by the informed consent of the participants under ethical approval number EBB 1.1-12/624.

### Replication cohorts for associations of Neandertal haplotypes

For additional analyses, we leveraged GWAS summary statistics for Neandertal haplotypes in NESDA, Biobank Japan, FinnGen [https://r5.finngen.fi/], deCode (extracted from Skov et al. [30]), and the Estonian Biobank (see above).

### Definition of Neandertal marker variants

We used previously inferred putative marker SNPs that tag tracts of Neandertal ancestry in the 1000 Genomes (phase 3, Table S2) [31, 32]. Putative introgressed Neandertal variants, referred to as aSNPs, were defined as being (i) absent in the 1,000 Genomes Yoruba population, the population demonstrated to show the lowest levels of Neandertal DNA in this cohort [31] (ii) present in homozygous state in either the Altai or Vindija Neandertal, two high coverage Neandertal genomes [10, 11] and (iii) present in at least one 1000 Genomes non-African individual. Aside from admixture, another genomic feature that could lead to a similar allele-sharing pattern is incomplete lineage sorting (ILS). However, because Neandertal admixture into modern humans is much more recent than the shared common ancestor of Neandertals and modern humans, shared variants that result from introgression are on haplotypes that are much longer than those on which variants resulting from ILS are found. We therefore required (iv) aSNPs to be on haplotypes that exceed the length expected under ILS.

### Testing for the proportional association strength to phenotypes

In order to quantify the association strength of archaic variants, we compared their number of significantly associated tag aSNPs to the number of frequency-matched non-archaic tag SNPs following similar approaches that have previously been used for such comparisons [21, 22, 32, 33]. These approaches implement measures to account for multiple differences between archaic and non-archaic variants that would otherwise potentially bias this analysis (see Supplementary Methods for a more detailed description).

First, due to the recent admixture ~55,000 years ago and the separation between modern humans and Neandertals before, introgressed Neandertal DNA is found on haplotypes of tens or even more than 100 thousand base pairs with several aSNPs in high linkage disequilibrium (LD). In order to account for the - on average - higher levels of LD for archaic variants compared to non-archaic variants, we generated sets of SNPs in LD of *r*^2^ > 0.5 and selected a random tag SNP. If the set contained aSNPs we annotated it as archaic sets and selected a random tag aSNP to represent the set. Sets without aSNPs were annotated as non-archaic and represented by a random tag SNP. Finally, SNPs without any other variants in LD were defined to be their own tag SNP. In the three cohorts we found 14,839 (UK Biobank), 12,111 (Biobank Japan) and 14,596 (NESDA) tag aSNPs.

We then calculated the number of significant tag aSNP associations based on varying *P* value cutoffs of 10^−3^, 10^−4^, 10^−5^, 10^−6^, 10^−7^ for the analyses in the Biobank Japan and UK Biobank cohorts and 10^−2^, 10^−3^ for the NESDA cohort. We chose these different cutoffs to account for trait-specific features in individual association analyses, such as heritability or prevalence of the tested trait in the cohort.

A second feature that is specific for aSNPs is its frequency distribution that is linked to the rather low prevalence of Neandertal DNA of ~2% compared to non-archaic variation in present-day non-African populations. In order to account for frequency-dependent differences between archaic and non-archaic variants and the subsequent differences in detection power we tested for a disproportionate number of tag aSNPs associations by comparing the number tag aSNP association to 1000 sets of frequency-matched non-archaic tag SNPs.

We then report the average of the 1000 ratios between the number of tag aSNP associations to the number of association in the random sets in the form of an odds ratio (OR) and empirical P values based on the location of the number of tag aSNP associations within the distribution of associations for the 1000 random sets. We applied this test to both individual phenotypes and groups of phenotypes. For the latter case we calculated the sum of the numbers of tag aSNP associations across a group of phenotypes and compared it to the sum of numbers of associations of 1000 random sets of frequency-matched non-archaic tag SNPs.

In order to account for multiple testing we generate false-discovery rates (FDR) using the *P* value correction approach by Benjamini–Hochberg.

Our approach is generally able to detect an enrichment of aSNPs above association *P* value cutoffs. As aSNPs at higher cut-off are typically rare, this method is better equipped to detect such enrichment results than alternative approaches, such as a comparison of the entire aSNP *P* value distribution compared to distributions of frequency-matched non-archaic variants. In addition, previous studies have pointed out that Neandertal DNA shows a lower prevalence in regions that are associated with higher functional capacity in the human genome [12, 34]. We therefore consider the results obtained in this study a rather conservative estimate in terms of their implications for phenotypic enrichments as random non-archaic variants are likely associated with an, on average, higher functional surrounding genomic content.

### Functional annotation of Neandertal variants

We defined loci to be Neandertal DNA risk loci if they contained aSNPs with an phenotype association *P* value below 5 × 10^−8^ and if these aSNPs were themselves the top association in a given genomic region or in linkage disequilibrium of *r*^2^ > 0.5 with the top associated SNP (Table S3). Frequencies for each candidate aSNP were calculated in 1000 Genomes populations (phase 3) [31]. For each of these aSNP associations we extracted archaic haplotypes with a range that was specified by location of other aSNPs with *r*^2^ > 0.5 with the candidate aSNP (Table S3). We explored GTEx (version 8) [35] for significant eQTLs that were linked to the candidate aSNP or other aSNPs associated with its archaic haplotype. Similarly, we tested whether any candidate aSNP or aSNPs associated with the candidate aSNPs’ haplotype were predicted to modify the amino acid sequence using the ENSEMBLs’ Variant Effect Predictor [36] by extracting all of the aSNPs that were annotated as ‘missense variant’.

## Results

### Diagnostic categories of CNS disorders do not show robust links to Neandertal DNA variants

In the UKBB, our analysis included GWAS summary statistics for 11 mental, behavioral and neurodevelopmental disorders (ICD10 codes F01-F99) and 21 diseases of the nervous system (ICD10 codes G00-G99). The GWAS data was generated based on 361,194 individuals and ~8.6 million SNPs with minor allele frequency (MAF) larger than 1% after QC filters on samples and genotypes. (see “Methods” for details).

We annotated putative introgressed Neandertal variants based on previously described marker SNPs, referred to as aSNPs [32]. We found and analyzed 197,250 such aSNP in the UK Biobank cohort. We then tested for a disproportionate number of aSNP associations for a given phenotype by calculating the number of LD-corrected tag aSNP associations and comparing it to the numbers of associations in 1000 random sets of frequency-matched and LD-corrected non-archaic tag SNPs (“Methods”).

We first applied this method to the two combined groups of disorders. Overall, consistent with the observation by Simonti et al. [21], we found an enrichment for introgressed Neandertal alleles associated with diseases of the nervous system (see Fig. 1c). However, this was only significant after FDR correction for the least conservative significance cut-off (OR > 1, *P* < 0.05 and FDR < 0.05 for *P* value cut-off 10^−3^). Averaged across all significance cutoffs, neither the group of mental, behavioral or neurodevelopmental disorders, nor the group of nervous system diseases codes showed significant enrichment of tag aSNP associations compared to frequency-matched non-archaic tag SNPs (see Fig. 1d). When considering disorders individually, only a few signals appeared, mostly for neuropathies (see Supplementary Materials).

As a complementary approach, we next explored medication prescriptions as a proxy for disease in the UKBB. Based on the classification of the World Health Organization, we annotated 626 medications with available data in the UK Biobank (Table S4). When testing whether the cumulative sum of tag aSNP associations across the 96 medications for CNS disorders (category N) differed between tag aSNPs and non-archaic tag SNPs, tag aSNPs showed over-proportional association numbers for one P value cut-off (Fig. 1c, OR > 1, *P* < 0.05 for *P* value cut-off 10^−7^, Table S1). When averaged across all significance cutoffs, CNS medication did not show significant associations with Neandertal DNA (see Fig. 1d). However, when breaking down CNS medications into subcategories, we did detect a significant signal for a link between Neandertal DNA and two classes of pain medications (see Supplementary Materials).

### Behavioral phenotypes with relevance for mental health show robust enrichment for Neandertal variants

When we explored Neandertal associations with related but “non-disease” behavioral phenotypes, signals became substantially stronger. Questionnaire data were available for numerous phenotypes related to mental health (47 questions), sleep (6), pain (17), smoking (33) and alcohol use (26). We again first quantified the cumulative numbers of tag aSNP associations across GWAS within each of the five groups. We found that GWAS of smoking (all *P* < 0.001 and FDR < 0.001, all OR > 1, Table S1), sleep traits (all *P* < 0.05 and 4 of 5 FDR < 0.05, all OR > 1) and alcohol intake (all *P* < 0.05 and 2 of 5 FDR < 0.05, all OR > 1) showed consistently larger numbers of associations with tag aSNPs when compared to their non-archaic counterparts (Table S1 and Fig. 1c), which were significant across all significance cut-offs. Consequently, these groups of traits showed significant enrichment when averaged across all significance cutoffs (Fig. 1d).

In order to identify the most likely driver of the enrichment results for these three groups we analyzed individual GWAS within each groups and found that six *smoking-related phenotypes* showed notable enrichments; including two describing the number of daily smoked cigarettes (all OR > 1, 9 of 10 *P* < 0.05 and 6 of 10 FDR < 0.05, Table S1) and another six defining smoking status (8 × *P* < 0.05 and 6 × FDR < 0.05, Table S1).

An additional six *alcohol GWAS* showed enrichments (all OR > 1 and *P* < 0.05 with one also FDR < 0.05). Four of these phenotypes characterized regular intake frequencies for various alcoholic beverages (Table S1), one defining the alcohol drinker status and another one specifying the habit of consuming alcohol during meals. Moreover, four significant phenotypes relate to *chronotype* and one to *sleep duration* (Table S1).

The combined group of *mental health GWAS* showed association enrichment results similar to alcohol, smoking, and sleeping habits for the two most relaxed P value cutoffs (OR > 1, *P* < 0.01 and FDR < 0.05 for 10^−3^ and 10^−4^). With 47 underlying GWAS, the group of ‘mental health’ combines the second largest number of individual GWAS within our tested groups. Among those, 14 GWAS showed at least one enrichment test with OR > 1 and *P* < 0.05 and were linked to various mood-related questions (Table S1). The most notable enrichment with odds ratios above 1, three instances of *P* < 0.05 and the only two cases of FDR < 0.05 was linked to the length of a depressive episode. The group of mental health phenotype associations also included one with the ‘Longest period of unenthusiasm/disinterest’ where a substantially lower number of associations with tag aSNPs was observed (OR < 1 and P < 0.05, Table S1).

Finally, while the combined group of *pain phenotypes* showed an average OR ≥ 1 for all tested *P* value cutoffs, none of these instances reached a significant level of differences between tag aSNP and non-archaic tag SNP associations (Fig. 1C, D, Tables S1 and S5). On the individual GWAS level, only three tests for pain, one related to each general pain, back pain, and knee pain, showed a larger number of tag aSNP associations with *P* < 0.05. One additional GWAS related to long term facial pain even showed lower numbers of associations with Neandertal variants (OR < 1 and *P* < 0.05, Table S1).

Taken together, these results indicate a robust enrichment of Neandertal variants associated with 5 groups of behavioral phenotypes in the UKBB: Alcohol consumption, smoking, mental health (specifically mood), chronotype/sleep, and pain. We would like to note that while our analysis of the cumulative numbers of Neandertal associations for groups of phenotypes was often well-powered, i.e. across *P* value cut-offs being based on tens of aSNPs (Table S1), our analyses of individual GWAS was repeatedly based on and driven by a few risk tag aSNPs. Thus, we further explored the robustness of associations of aSNPs in additional cohorts.

### A Neandertal haplotype is associated with smoking across different genomic cohorts

**Fig. 2:**
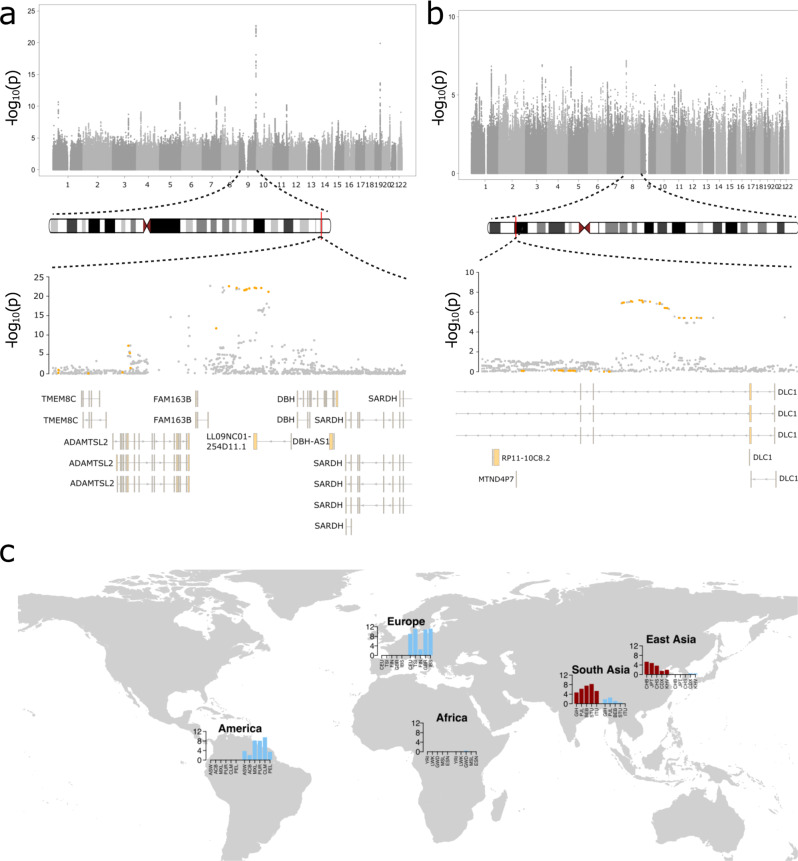
Top smoking risk loci in UK Biobank and Biobank Japan are linked to Neandertal DNA. **a**, **b** Manhattan plots for the ‘Current smoking status’ GWAS in the UK Biobank (**a**) and ‘Ever smoked’ GWAS in the Biobank Japan (**b**) are shown in the respective upper parts of the panels. A magnified view centered (+−100,000 base pairs) around the top genome-wide associations on chromosome 9 (**a**, chr9:136,363,019–136,563,019, *P* = 2.7 × 10^−23^) and chromosome 8 (**b**, chr8:12,889,111–13,689,111, *P* = 6.7 × 10^−8^) are displayed in the lower parts of **a** and **b**. Here, aSNPs are highlighted in orange. Overlapping genes and their gene models are shown in the lower part of the panel. **c** The frequency of the archaic alleles for the two top associated aSNPs (dark red: *rs113382419*, chr9:136,463,019; and light blue: *rs76591447*, chr8:13,289,111) in present-day 1000 Genomes continental populations are displayed.

We found 27 instances linked to 18 independent Neandertal loci that showed genome-wide significant association (*P* < 5 × 10^−8^), with aSNPs showing the top association or being in high linkage disequilibrium with the lead SNP in a given region (Table S3). Eighteen of the 27 associations were linked to smoking and sleeping patterns, suggesting that particularly for these phenotypes, Neandertal DNA contributes large effect variants. We also noted that two risk-increasing GWAS lead variants for smoking status in both the UK Biobank (lead aSNP: *rs113382419*, chr9:136,463,019_C/A, *P* = 2.7 × 10^−23^, *β* = 0.02, archaic AF = 11.2%) and Biobank Japan (lead aSNP: *rs76591447*, chr8:13,289,111_C/G, *P* = 6.7 × 10^−8^, *β* = −0.02, archaic AF = 3.2%) were linked to aSNPs (Fig. 2 and Table S3). Both lead aSNPs showed population-specific frequency differences, a pattern that was highly prevalent among other high-risk Neandertal variants as well (Table S3).

Next, we explored whether any of the two smoking risk variants showed comparable association results in other cohorts as well. We were able to assess the association scores for the UK Biobank risk variant (or aSNPs in close proximity) in four additional cohorts: Here, we leveraged GWAS summary statistics aSNP *rs113382419* (which was significantly associated with smoking status in the UBB) or - if not available - the aSNPs *rs3025343* and *rs1985381* which are linked to the same archaic haplotype (Table S3) (Table 1). In NESDA we explored our generated GWAS on the ‘ever smoked’ item (*P* = 2.2 × 10^−4^ for *rs1985381*); in FinnGen [https://r5.finngen.fi/] we queried the ‘R5 Smoking’ item (*P* = 0.004 for *rs113382419*); deCode summary statistics for the ‘current vs former smokers’ GWAS were extracted from Skov et al. [30] (*P* = 2.4 × 10^−7^ for *rs3025343*) and in the Estonian Biobank we scanned a GWAS on the ‘ever smoked’ item (*P* = 0.008 for *rs113382419*). Despite the fact that the smoking-related phenotypes in these cohorts did not exactly match the definition of smoking status in UK Biobank, we still found significant associations (*P* < 0.05, Table 1) with aSNPs related to the archaic haplotype of the UK Biobank risk aSNP: NESDA (‘ever smoked’, *P* = 2.2 × 10^−4^, *rs1985381*), FinnGen (R5 Smoking item, *P* = 0.004, *rs113382419*), deCode (‘current vs former smokers’, *P* = 2.4 × 10^−7^, *rs3025343*) and the Estonian Biobank (‘ever smoked’, *P* = 0.008, *rs113382419*).

**Table 1:** GWAS summary statistics for Neandertal smoking risk allele.

	Phenotype	aSNP	*P*	*β*
UK Biobank	Current smoking status	*rs113382419*	1.6 × 10^−19^	0.01
NESDA	Ever smoked	*rs1985381*	2.2 × 10^−4^	1.24
FinnGen	Smoking item R5	*rs113382419*	4.0 × 10^−3^	0.29
Estonian Biobank	Ever smoked	*rs113382419*	7.9 × 10^−3^	0.05
deCode	Current vs former smokers	*rs3025343*	2.4 × 10^−7^	1.14

GWAS summary statistics of smoking-related phenotypes from five cohorts. Displayed are the association statistics for the aSNP *rs113382419* or aSNPs associated with the same archaic haplotype. Note that different association models and software have been used across cohorts (see “Methods”) and hence, effect sizes are not directly comparable.

### Frequency and biological context of Neandertal risk loci

Importantly, 11 of the 18 Neandertal risk loci are linked to aSNPs with a frequency that was among the top 5% of aSNPs in at least one 1,000 Genomes population, including associations with all ten sleep-related traits, four of the five mental health phenotypes and two of the smoking habits. A particularly extreme example were aSNPs in the region of chr5:151,756,407–151,976,244, (association with chronotype and ‘getting up in the morning’) with frequencies between 21.5 and 55.2% in present-day Europeans, South Asians and Americans, putting them within the top 1% in 14 out of 15 of these populations.

**Fig. 3:**
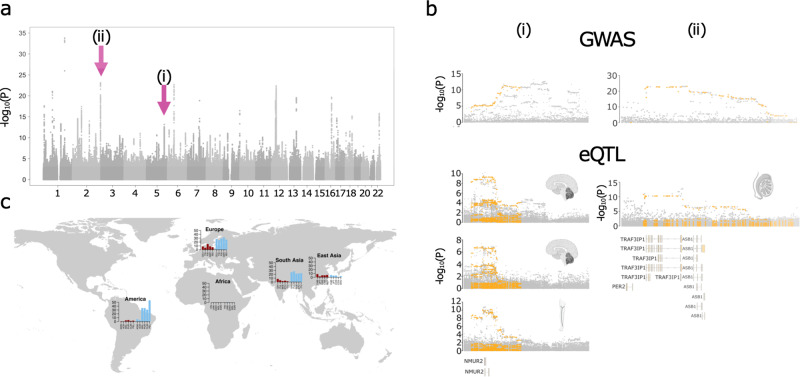
Neandertal DNA contributes to chronotype risk loci. **a** Manhattan plot for a Chronotype GWAS in the UK Biobank. **b** Magnified views of the association (*y*-axis, −log_10_ transformation of the association *p* value) score with chronotype on chromosomes 5 (left, chr5:151,589,813–152,689,813) and 2 (right, chr2:239,173,478–239,573,478) are shown on the top part of each panel. aSNPs are highlighted in orange. The lower part of each panel shows gene expression associations in GTEx tissues for the respective regions (eQTL −log [10] transformed association *P* values for—from top—Cerebellar_Hemisphere, Cerebellum and Tibial Nerve and Testis. Models of overlapping gene are illustrated at the bottom. **c** The frequency of the top associated aSNPs from the two illustrated regions (rs76939124, chr2:239,223,478 in red and *rs4958550*, chr5:151,889,813 in blue) across 1000 Genomes populations.

The aSNPs at this locus were associated with modified expression of three genes (*GLRA1, LINC01933* and *NMUR2*) in two brain regions and nerve tissue (Fig. 3 and Table S3). Archaic SNPs at another seven loci with links to four additional sleeping-related aSNP association, and one association for each, pain, smoking, and mental health showed regulatory effects as well in various tissues including arteries, testis, thyroid, muscle spleen, and ovaries (Table S3). In addition, we also found evidence for associated aSNPs affecting the amino acid sequences two genes; *SCML4* (rs117914882, chr6:108,076,801_T/C, ‘period of unenthusiasm/disinterest’, archaic AF < 1%) and *CHRNA5* (rs76071148, chr15:78,885,574_T/A, ‘Cigarette consumption per day’, archaic AF = 27.8%, Table S3).

### Analysis of overall Neandertal enrichment in independent cohorts

Finally, we explored Neandertal enrichment of phenotype associations in two independent cohorts of diverse ancestry. For this purpose, the Netherlands Study of Depression and Anxiety (NESDA) and the Japanese Biobank provided adequate genetic coverage to analyze tag aSNPs and sufficient depth in the behavioral data.

**Fig. 4:**
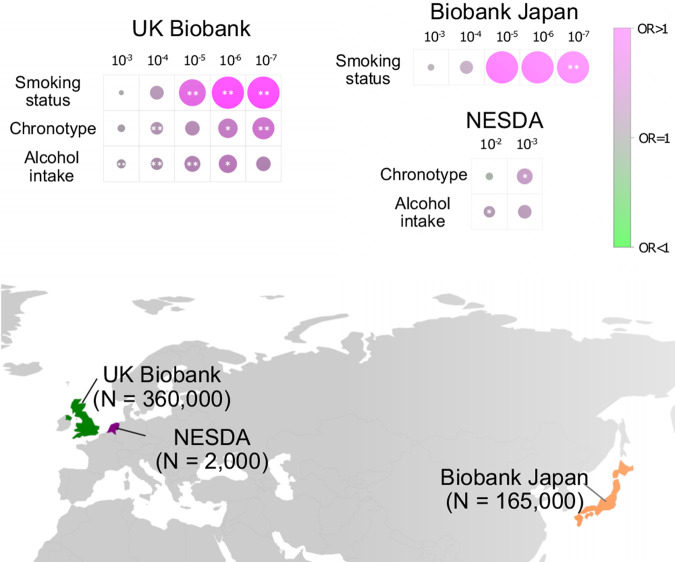
Analysis of Neandertal DNA associations in the Biobank Japan and Netherlands Study of Depression and Anxiety (NESDA). The three phenotype groups with the largest over-proportional Neandertal DNA associations in the UK Biobank cohort were smoking, sleep, and alcohol. The individual phenotypes in each group that showed the largest contributions to the respective groups’ enrichment results were smoking status, chronotype, and alcohol intake (top left). For these three phenotypes the odds ratio, reflecting the ratio between the number of tag aSNP associations and the average number of associations in 1000 sets of frequency-matched non-archaic tag SNPs is displayed (color and size indicate the average log odds ratio and direction). Using the same method the three phenotypes were among the phenotypes that were tested for the proportional number of Neandertal DNA associations in NESDA (results for the significant phenotypes of chronotype and alcohol intake are shown on the right). In addition, four smoking phenotypes were tested in Biobank Japan and the results for smoking status— one of the phenotypes with a significant enrichment result is shown in the top right. A map illustrating the host countries of each cohort and the sample sizes used for this analysis are displayed in the lower part of the figure.

In NESDA, we were able to probe eight behavioral phenotypes, one for smoking, one for alcohol consumption, two sleep-related phenotypes and four mental health phenotypes (Table S1). We applied the same enrichment analysis as in the UKBB, but - due to the reduced association power because of the substantially lower sample sizes in NESDA (*N* = 1842–2261)—we adjusted our *P* value cutoffs to 10^−2^, 10^−3^ (see “Methods”). Out of the 8 tested phenotypes, we found two instances with substantially larger numbers of tag SNP associations: Alcohol intake (OR > 1, *P* < 0.05, *P* value cut-off 10^−2^) and chronotype (OR > 1, *P* < 0.05, *P* value cut-off 10^−3^, Fig. 4 and Table S1).

Both the UK Biobank and NESDA cohorts include individuals of predominantly European ancestry. However, risk loci for complex traits have often been associated with population-specific genetic variants, a phenomenon that has also been observed for Neandertal DNA [32, 37]. In order to explore to which extent our results can be translated to cohorts of non-European ancestry, we explored summary statistics from the Biobank Japan. The Biobank provides GWAS summary statistics for four smoking traits derived from ~165,000 individuals with information about smoking status, daily cigarette consumption and age of onset [27]. Again, applying our enrichment method for tag aSNP (Methods) we found that a GWAS for smoking status showed a substantially larger number of Neandertal variants (FDR < 0.05, *P* value cut-off 10^−7^).

Taken together, despite the substantially lower power in NESDA, and the different ancestry in the Japan Biobank, we observed overall Neandertal enrichment for behavioral phenotypes of smoking, alcohol consumption, and chronotype.

## Discussion

In this study, we investigated the association strength of introgressed Neandertal DNA variants with neuropsychiatric disorders, nervous system medications (as a proxy for disease) and various behavioral (non-disease) phenotypes. Overall, while we found no associations with disease categories, there was an enrichment for associations of smoking and alcohol intake, pain and chronotype with Neandertal variants in the largest cohort (the UKBB). Intriguingly, most of the enriched behavioral phenotypes closely resemble endophenotypes that are often strongly correlated with neuropsychiatric diseases, also on a genetic level. For example, recent work has suggested that there is a potentially genetic causal link between chronotype and odds of developing depressive symptoms [38] and similar findings have been reported for smoking [39] or alcohol abuse [40] and depression. In addition to smoking and alcohol intake, Neandertal DNA also showed significantly higher levels of association with pain and pain medications.

Our findings that introgressed variants are not enriched in psychiatric and neurological diagnostic categories are in line with a recent broad analysis across a wide range of phenotypes by McArthur et al. that showed very limited associations of Neandertal variants with disease in modern humans beyond dermatological or immune-mediated disorders [34]. The study by McArthur et al was conducted simultaneously to ours in the publicly available UK Biobank cohort and employed a complementary approach to assess the relative levels of heritability for Neandertal-introgressed variants. Consistent with our results, this study also identifies individual phenotypes related to smoking and sleep that show enrichment for Neandertal variants on heritability. We note that McArthur et al restricted their analysis to Neandertal variants with a frequency above 5%, a cut-off that removes more than 50% of the variants we include in our study (frequency cut-off >1%). Given that the variants above 5% are likely not a random subset the overlap of enrichment results between both studies is not necessarily expected and supports the robustness of the results. In addition, our approach of combining GWAS summary statistics from individual GWAS from a group of phenotypes allows us to determine that the overall numbers of associations with these three groups of phenotypes is unexpectedly high. In addition, our analysis demonstrates that some of these phenotypes show comparable enrichment results in the NESDA and the Biobank Japan as well. The enrichment of associations with traits such as chronotype, pain, alcohol and tobacco use rather than diagnostic disease categories may thus reflect adaptations. For example, Neandertal variants could persist in modern humans due to their neutral or even potentially advantageous effects at some point during recent evolution. Some previous studies have suggested that evolutionary forces such as positive selection [41] and Neandertal admixture [42, 43] may be linked to mental disorders such as Schizophrenia. A more recent study subsequently demonstrated that when accounting for purifying selection there is no evidence for a significant Neandertal contribution [44]. Genomic footprints of complex traits under purifying selection include an altered LD structure, fewer high effect variants and in general phenotype-associated variants at on average lower allele frequencies. Our enrichment method is designed to account for LD differences for different variants in the genome, and by applying multiple significance cutoffs and comparisons to frequency-matched non-archaic background sets of variants is also equipped to consider the frequency distribution of associated variants. Consequently, we do not find an enrichment result with Schizophrenia. In general, the lack of an enrichment of Neandertal associations with psychiatric and neurological diagnostic categories by our study and McArthur et al. is consistent with previous studies that have shown that many complex traits, and in particular diseases, are targets of purifying selection [45, 46].

In addition, the design of our enrichment method was able to address both the - on average - higher levels of LD and lower allele frequencies for aSNPs compared to non-archaic variants; two major genomic differences that have a major impact on association analyses. Attempts to control for other genomic factors, like distance to genes or other functional elements in the genome would likely introduce other unwanted and uncontrollable biases. However, given that previous analyses have demonstrated that Neandertal DNA is showing signals of depletion around functional gene groups [12, 34], makes our current setup rather conservative as it shouldn’t result in an artificial enrichment result due to functional genome context.

Taken together, these studies and our current results suggest that Neandertal variants are probably not directly linked to diagnostic entities of mental disorders but may have some indirect links, e.g. via behavioral phenotypes such as smoking, pain, or sleep, which in turn are linked to diseases.

Interestingly, genomic regions that differ most between Neandertals and modern humans, including regions where no introgressed archaic DNA can be detected in people today [14, 47] have previously been linked to brain-related genes [14, 47]. What exactly the evolutionary advantages of such phenotypes might have been, however, remains speculative at this point. Elevated frequencies of sleep-associated Neandertal variants are suggestive of them having been targets of adaptive processes. Sleep patterns and other behavioral phenotypes can be linked to circadian rhythm which in turn can be linked in differences of UV light exposure. If Neandertals were adapted to the UV light regime in Europe and Western Asia and contributed these adaptive alleles to modern humans, this may explain our enrichment results and in the case of sleep-related phenotypes suggest that they might have helped modern human adaptation to these new environments. With regard to pain, a previous analysis has indicated that Neandertal-introgressed DNA near the *SCN9A* gene in modern humans is associated with lower pain thresholds and revealed a putative biological mechanism by implicating amino acid substitutions introduced by introgressed Neandertal variants in the sodium channel Nav1.7 protein [48]. With regard to smoking or alcohol, evolutionary origins have been postulated for addiction, suggesting a co-evolution of the human brain, reward-seeking, and psychotropic substances [49]. In line with this idea, “thrill-seeking” was one of the phenotypes where we observed Neandertal associations in the UKBB. An alternative—non-exclusive—hypothesis could be that this reflects self-medication for pain [50], so that all of these associations (as well as the reported link with some pain medications) may be driven by the same selection processes.

Of note, in some instances, the enrichment results of individual phenotypes were primarily driven by Neandertal variants that have reached genome-wide significance or may even—as in the case of smoking status in both the UK Biobank and Biobank Japan—represent the strongest association genome-wide. We were also able to show evidence that the UK Biobank risk variant is showing strong associations with similar smoking traits in four additional cohorts. However, the variability of the association *P* values in these four cohorts (ranging between 0.006 and 2.4 × 10^−7^) also suggest that particularly for single traits with few Neandertal risk variants it likely remains hard to replicate an overall enrichment of Neandertal associations. These observations also highlight why our initial analysis of groups of phenotypes is important as it provides us with enrichment results that are based on larger sets of Neandertal variants and are therefore likely providing a more robust phenotype target. Nevertheless, given that our LD-corrected tag aSNPs make up only around 1% of tag SNPs (with MAF > 1%) in UK Biobank and Biobank Japan, we still consider the enrichment results of smoking status in these cohorts as informative, as they pointed out instances of top risk variants of Neanderthal ancestry. The presence of population-specific Neandertal risk variants for this trait may also directly implicate certain biological pathways in the observed genetic associations. In general, the majority of these and other genome-wide significant associations (18/27) were related to smoking and sleeping. Some of the association with sleep patterns were linked to Neandertal variants that have reached exceptionally high frequencies compared to other introgressed DNA in some present-day populations, suggesting that they may have been positively selected at some point in the past (Table S3). We also show that some of these variants are linked to well-established candidate genes for smoking status. For example, the aSNP *rs117914882*, which was associated with an increased ‘period of unenthusiasm/disinterest’ in our analysis of the UKBB, modifies an arginine to a glycine in the protein sequence of SCML4. This change is predicted as ‘probably damaging’ by PolyPhen [51] (Table S3). SCML4 has previously been linked to stress reactions in mice and modifications to its protein structure might therefore further contribute to related effects in mood phenotypes [52]. Another protein sequence altering aSNP was rs76071148 (archaic haplotype chr15:78,803,937–78,957,720), where the archaic allele A changes a histidine—the majority amino acid in present-day people—to a glutamine in *CHRNA5* (Table S3). This modification is also classified as ‘possibly damaging’ by PolyPhen. *CHRNA5* has been linked in several studies to smoking and various smoking risk factors [53–57].

Some limitations of our study have to be considered. For example, in our methodological approach, we have made a number of decisions that we feel best address the challenges of comparisons for Neandertal DNA associations with disease categories and behavioral traits. We acknowledge that other approaches such as e.g. SNP-based heritability enrichment [58] could serve as an alternative to address these questions. We decided to use different cut-offs to account for trait-specific features like the aforementioned different levels of background selection. It is likely that only a small set of strongly associated Neandertal variants are separating modern archaic human-associated variation. We therefore consider the cut-off-based approach provides more power over a comparison of, for example, entire *P* value distributions. Thus, we believe our results robustly demonstrate that we are capable of picking up instances where a few strongly associated Neandertal variants are significantly influencing the association landscape of multiple behavioral phenotypes.

In conclusion, our study provides an example of how evolutionary information can help interpreting the origin and genetic components of behavioral phenotypes. We show that while Neandertal DNA shows over-proportional numbers of associations with endophenotypes, this enrichment does not translate to disease. This evolutionary knowledge may help to decipher the environmental factors that shaped phenotypes.

## Supplementary information

Supplementary Methods

Supplementary Tables Legends

Supplementary Tables

## References

[1] Flint J, Kendler KS (2014). The genetics of major depression. Neuron.

[2] Schizophrenia Working Group of the Psychiatric Genomics Consortium. (2014). Biological insights from 108 schizophrenia-associated genetic loci. Nature.

[3] Wray NR, Ripke S, Mattheisen M, Trzaskowski M, Byrne EM, Abdellaoui A (2018). Genome-wide association analyses identify 44 risk variants and refine the genetic architecture of major depression. Nat Genet.

[4] Hyde CL, Nagle MW, Tian C, Chen X, Paciga SA, Wendland JR (2016). Identification of 15 genetic loci associated with risk of major depression in individuals of European descent. Nat Genet.

[5] Levey DF, Stein MB, Wendt FR, Pathak GA, Zhou H, Aslan M (2021). Bi-ancestral depression GWAS in the Million Veteran Program and meta-analysis in >1.2 million individuals highlight new therapeutic directions. Nat Neurosci.

[6] Howard DM, Adams MJ, Clarke T-K, Hafferty JD, Gibson J, Shirali M (2019). Genome-wide meta-analysis of depression identifies 102 independent variants and highlights the importance of the prefrontal brain regions. Nat Neurosci.

[7] Nesse RM. Evolutionary psychology and mental health. In: The handbook of evolutionary psychology; Hoboken, NY, USA: John Wiley & Sons, Inc.; 2015. p. 1–20.

[8] Provenzano F, Deleidi M. Reassessing neurodegenerative disease: immune protection pathways and antagonistic pleiotropy. Trends Neurosci. 2021. 10.1016/j.tins.2021.06.006. 10.1016/j.tins.2021.06.006 34284880

[9] Sankararaman S, Patterson N, Li H, Pääbo S, Reich D (2012). The date of interbreeding between Neandertals and modern humans. PLoS Genet.

[10] Prüfer K, Racimo F, Patterson N, Jay F, Sankararaman S, Sawyer S (2014). The complete genome sequence of a Neanderthal from the Altai Mountains. Nature.

[11] Prüfer K, de Filippo C, Grote S, Mafessoni F, Korlević P, Hajdinjak M (2017). A high-coverage Neandertal genome from Vindija Cave in Croatia. Science.

[12] Sankararaman S, Mallick S, Dannemann M, Prüfer K, Kelso J, Pääbo S (2014). The genomic landscape of Neanderthal ancestry in present-day humans. Nature.

[13] Meyer M, Kircher M, Gansauge M-T, Li H, Racimo F, Mallick S (2012). A high-coverage genome sequence from an archaic Denisovan individual. Science.

[14] Vernot B, Tucci S, Kelso J, Schraiber JG, Wolf AB, Gittelman RM (2016). Excavating Neandertal and Denisovan DNA from the genomes of Melanesian individuals. Science.

[15] Fu Q, Posth C, Hajdinjak M, Petr M, Mallick S, Fernandes D (2016). The genetic history of Ice Age Europe. Nature.

[16] Harris K, Nielsen R (2016). The Genetic Cost of Neanderthal Introgression. Genetics.

[17] Petr M, Pääbo S, Kelso J, Vernot B (2019). Limits of long-term selection against Neandertal introgression. Proc Natl Acad Sci USA.

[18] Dannemann M, Racimo F (2018). Something old, something borrowed: admixture and adaptation in human evolution. Curr Opin Genet Dev.

[19] Gittelman RM, Schraiber JG, Vernot B, Mikacenic C, Wurfel MM, Akey JM (2016). Archaic hominin admixture facilitated adaptation to Out-of-Africa environments. Curr Biol.

[20] Sankararaman S, Mallick S, Patterson N, Reich D (2016). The combined landscape of Denisovan and Neanderthal ancestry in present-day humans. Curr Biol.

[21] Simonti CN, Vernot B, Bastarache L, Bottinger E, Carrell DS, Chisholm RL (2016). The phenotypic legacy of admixture between modern humans and Neandertals. Science.

[22] Dannemann M, Kelso J (2017). The contribution of Neanderthals to phenotypic variation in modern humans. Am J Hum Genet.

[23] Cai N, Choi KW, Fried EI (2020). Reviewing the genetics of heterogeneity in depression: operationalizations, manifestations and etiologies. Hum Mol Genet.

[24] Bycroft C, Freeman C, Petkova D, Band G, Elliott LT, Sharp K (2018). The UK Biobank resource with deep phenotyping and genomic data. Nature.

[25] Penninx BWJH, Beekman ATF, Smit JH, Zitman FG, Nolen WA, Spinhoven P (2008). The Netherlands Study of Depression and Anxiety (NESDA): rationale, objectives and methods. Int J Methods Psychiatr Res.

[26] Nagai A, Hirata M, Kamatani Y, Muto K, Matsuda K, Kiyohara Y (2017). Overview of the BioBank Japan Project: Study design and profile. J Epidemiol.

[27] Matoba N, Akiyama M, Ishigaki K, Kanai M, Takahashi A, Momozawa Y (2019). GWAS of smoking behaviour in 165,436 Japanese people reveals seven new loci and shared genetic architecture. Nat Hum Behav.

[28] Marchini J, Howie B (2010). Genotype imputation for genome-wide association studies. Nat Rev Genet.

[29] Leitsalu L, Haller T, Esko T, Tammesoo M-L, Alavere H, Snieder H (2015). Cohort profile: Estonian Biobank of the Estonian Genome Center, University of Tartu. Int J Epidemiol.

[30] Skov L, Coll Macià M, Sveinbjörnsson G, Mafessoni F, Lucotte EA, Einarsdóttir MS (2020). The nature of Neanderthal introgression revealed by 27,566 Icelandic genomes. Nature.

[31] Auton A, Brooks LD, Durbin RM, Garrison EP, Kang HM, 1000 Genomes Project Consortium (2015). A global reference for human genetic variation. Nature.

[32] Dannemann M. The population-specific impact of Neandertal Introgression on Human Disease. Genome Biol Evol. 2021;13. 10.1093/gbe/evaa250. 10.1093/gbe/evaa250 PMC7851588 33247712

[33] Quach H, Rotival M, Pothlichet J, Loh Y-HE, Dannemann M, Zidane N (2016). Genetic adaptation and Neandertal Admixture Shaped the Immune System of Human Populations. Cell.

[34] McArthur E, Rinker DC, Capra JA (2021). Quantifying the contribution of Neanderthal introgression to the heritability of complex traits. Nat Commun.

[35] GTEx Consortium, Laboratory, Data Analysis & Coordinating Center (LDACC)—Analysis Working Group, Statistical Methods groups—Analysis Working Group, Enhancing GTEx (eGTEx) groups, NIH Common Fund, NIH/NCI, et al. Genetic effects on gene expression across human tissues. Nature. 2017;550:204–13. 10.1038/nature24277 PMC5776756 29022597

[36] McLaren W, Gil L, Hunt SE, Riat HS, Ritchie GRS, Thormann A (2016). The Ensembl variant effect predictor. Genome Biol.

[37] Vernot B, Akey JM (2014). Resurrecting surviving Neandertal lineages from modern human genomes. Science.

[38] O’Loughlin J, Casanova F, Jones SE, Hagenaars SP, Beaumont RN, Freathy RM, et al. Using Mendelian Randomisation methods to understand whether diurnal preference is causally related to mental health. Mol Psychiatry. 2021. 10.1038/s41380-021-01157-3. 10.1038/s41380-021-01157-3 PMC8760058 34099873

[39] Wootton RE, Richmond RC, Stuijfzand BG, Lawn RB, Sallis HM, Taylor GMJ (2020). Evidence for causal effects of lifetime smoking on risk for depression and schizophrenia: a Mendelian randomisation study. Psychol Med.

[40] Polimanti R, Peterson RE, Ong J-S, MacGregor S, Edwards AC, Clarke T-K (2019). Evidence of causal effect of major depression on alcohol dependence: findings from the psychiatric genomics consortium. Psychol Med.

[41] Song W, Shi Y, Wang W, Pan W, Qian W, Yu S (2021). A selection pressure landscape for 870 human polygenic traits. Nat Hum Behav.

[42] Srinivasan S, Bettella F, Mattingsdal M, Wang Y, Witoelar A, Schork AJ (2016). Genetic markers of human evolution are enriched in schizophrenia. Biol Psychiatry.

[43] Gregory MD, Eisenberg DP, Hamborg M, Kippenhan JS, Kohn P, Kolachana B (2021). Neanderthal-derived genetic variation in living humans relates to schizophrenia diagnosis, to psychotic symptom severity, and to dopamine synthesis. Am J Med Genet B Neuropsychiatr Genet.

[44] Pardiñas AF, Holmans P, Pocklington AJ, Escott-Price V, Ripke S, Carrera N (2018). Common schizophrenia alleles are enriched in mutation-intolerant genes and in regions under strong background selection. Nat Genet.

[45] Gazal S, Finucane HK, Furlotte NA, Loh P-R, Palamara PF, Liu X (2017). Linkage disequilibrium-dependent architecture of human complex traits shows action of negative selection. Nat Genet.

[46] O’Connor LJ, Schoech AP, Hormozdiari F, Gazal S, Patterson N, Price AL (2019). Extreme polygenicity of complex traits is explained by negative selection. Am J Hum Genet.

[47] Schaefer NK, Shapiro B, Green RE. An ancestral recombination graph of human, Neanderthal, and Denisovan genomes. Sci Adv. 2021;7. 10.1126/sciadv.abc0776. 10.1126/sciadv.abc0776 PMC8284891 34272242

[48] Zeberg H, Dannemann M, Sahlholm K, Tsuo K, Maricic T, Wiebe V (2020). A Neanderthal sodium channel increases pain sensitivity in present-day humans. Curr Biol.

[49] Saah T (2005). The evolutionary origins and significance of drug addiction. Harm Reduct J.

[50] Thompson T, Oram C, Correll CU, Tsermentseli S, Stubbs B (2017). Analgesic effects of alcohol: a systematic review and meta-analysis of controlled experimental studies in healthy participants. J Pain.

[51] Adzhubei I, Jordan DM, Sunyaev SR. Predicting functional effect of human missense mutations using PolyPhen-2. Curr Protoc Hum Genet. 2013; Chapter 7: Unit7.20. https://currentprotocols.onlinelibrary.wiley.com/doi/10.1002/0471142905.hg0720s76. 10.1002/0471142905.hg0720s76 PMC4480630 23315928

[52] Flati T, Gioiosa S, Chillemi G, Mele A, Oliverio A, Mannironi C (2020). A gene expression atlas for different kinds of stress in the mouse brain. Sci Data.

[53] Ware JJ, van den Bree M, Munafò MR (2012). From men to mice: CHRNA5/CHRNA3, smoking behavior and disease. Nicotine Tob Res.

[54] Cushing KC, Chiplunker A, Li A, Sung YJ, Geisman T, Chen L-S (2018). Smoking Interacts With CHRNA5, a Nicotinic Acetylcholine Receptor Subunit Gene, to Influence the Risk of IBD-Related Surgery. Inflamm Bowel Dis.

[55] Hartz SM, Short SE, Saccone NL, Culverhouse R, Chen L, Schwantes-An T-H (2012). Increased genetic vulnerability to smoking at CHRNA5 in early-onset smokers. Arch Gen Psychiatry.

[56] Lassi G, Taylor AE, Timpson NJ, Kenny PJ, Mather RJ, Eisen T (2016). The CHRNA5–A3–B4 gene cluster and smoking: from discovery to therapeutics. Trends Neurosci.

[57] Jensen KP, DeVito EE, Herman AI, Valentine GW, Gelernter J, Sofuoglu M (2015). A CHRNA5 smoking risk variant decreases the aversive effects of nicotine in humans. Neuropsychopharmacology.

[58] Finucane HK, Bulik-Sullivan B, Gusev A, Trynka G, Reshef Y, Loh P-R (2015). Partitioning heritability by functional annotation using genome-wide association summary statistics. Nat Genet.

