# Supplementary material for: Neandertal introgression partitions the genetic landscape of neuropsychiatric disorders and associated behavioral phenotypes
Source: Transl Psychiatry. 2022 Oct 5;12:433. doi: 10.1038/s41398-022-02196-2 (PMC9534885; doi:10.1038/s41398-022-02196-2)
Supplement: Supplementary file 1 — Supplementary Methods [file 41398_2022_2196_MOESM1_ESM.docx]

## **Supplementary Methods**

#### UK Biobank and its Neandertal DNA content

Summary statistics for genome-wide summary analysis (GWAS) from the UK Biobank ^24^ were obtained from the Neale lab [http://www.nealelab.is/uk-biobank/]. A detailed description of the analysis can be found at http://www.nealelab.is/blog/2017/9/11/details-and-considerations-of-the-uk-biobank-gwas and http://www.nealelab.is/blog/2019/10/24/updating-snp-heritability-results-from-4236-phenotypes-in-uk-biobank. In summary, GWAS have been conducted using 361,194 biobank individuals that have passed quality control filters. Based on these filters samples with non-British ancestry, are related, had sex chromosome aneuploidies or withdrawn from the biobank were excluded. Genotypes have been generated using two custom arrays and additional variants have been imputed using the 1,000 Genomes Project ^31^, the Haplotype Reference Consortium ^59^ and the UK10K cohort ^60^. The genotype data included ∼10.8 million SNPs with a minor allele frequency >0.1%, a Hardy–Weinberg equilibrium P value >1 × 10^−10^ and an INFO score, provided by the UK Biobank larger than 0.8. The association studies have been performed using the PHESANT software ^61^ and included as covariates the first 20 PCs, sex, age, age^2^, sex × age, and sex × age^2^ for diseases with male and female samples and only the nonsex covariates of this model for sex-specific diseases. We further restricted our analyses on 8,603,515 bi-allelic SNPs with a minor allele frequency larger than 1% in the cohort. Among those variants we identified 197,250 aSNPs.

#### Biobank Japan and its Neandertal DNA content

We used publicly available summary statistics for four smoking GWAS from Biobank Japan ^27^. These GWAS have been conducted in ~200,000 individuals from the Biobank Japan cohort, with up to 165,436 individuals per GWAS. Included individuals have been between the ages of 20 and 89 and all been diagnosed with at least one of 45 diseases. Genotyping data has been generated using three custom arrays and SNPs characterized on multiple arrays included in the analysis. Samples with a high call rate (>0.98), unrelated and of East Asian ancestry have been kept. SNPs with a frequency below 0.005, call rate < 0.99 have been excluded. Imputation has been performed using 275 East Asians from the 1,000 Genomes and SNPs with a minor allele frequency below 1% and Hardy Weinberg equilibrium P < 1x10^-6^ were excluded. We included 5,826,586 of those SNPs into our analysis. We identified a total 62927 aSNPs among this set.

The smoking phenotypes have been defined as (1) ever versus never smokers, (2) smoking cessation, (3) age of smoking initiation and (4) quantity of smoking. The association analyses have been analyzed using a linear mixed model using phenotype-specific combinations of covariates including age, age^2^, sex and the status across 45 diseases.

#### NESDA GWAS generation and its Neandertal DNA content

Methods for biological sample collection and DNA extraction have been described previously ^62^ as well as quality control and imputation pipelines ^63^. Briefly, 95% of the samples were genotyped on the Affymetrix 6.0 Human SNP array and the remaining on the Perlegen-Affymetrix 5.0 array. After platform-specific QC the missing SNP genotypes between each platform were imputed using the GONL (Genome of the Netherlands) ^64–66^ reference panel and then merged, followed by additional more stringent QC.

This cross-platform GONL imputed dataset was used to identify ancestry outliers, defined based on Principal Components Analysis (PCA) by projecting 10 PCs from 1,000 Genomes reference set populations on the NTR cross-platform imputed data using the SMARTPCA program as described earlier ^67,68^. Individuals with PC values located outside of the range of European and/or British populations were defined as outliers. Upon exclusion of outliers, 10 PCs were recomputed for cross-platform imputed data to capture the variation within the Netherlands.

The SNPs from the cross-platform GONL imputed dataset (~1.3M) were used for a second round of imputations to the 1000G Phase 3 ^31^ all ancestries reference panel using the Michigan Imputation Server ^69^.

Finally, the cross-platform imputed dataset was used to build a relationship matrix measuring genetic similarity using GCTA ^70^, which was pruned at 0.05 threshold in order to retain unrelated participants. Based on this genotype data we included 8,657,974 SNPs with a minor allele frequency larger than 1% for our analyses, 198,285 of which were aSNPs.

Genome-wide association analyses assuming an additive model were carried out using SNPTEST ^28^. All analyses were adjusted for age, sex and 10 ancestry-informative PCs. Association analyses related to alcohol intake were performed consistently with previous GWAS ^71,72^ including NESDA. The phenotype for this analyses was defined as the sex-specifc residuals (adjusted for age, age^2^, weight and 10 PCs) of (log_10_)gr/day alcohol intake.

#### Estonian GWAS generation and its Neandertal DNA content

Genotyping and imputation

DNA samples from the Estonian Biobank (EstBB) were genotyped at the Core Genotyping Lab of the Institute of Genomics, University of Tartu using the Illumina Global Screening Arrays (GSAv1.0, GSAv2.0, and GSAv2.0_EST). 200,000 samples were genotyped and PLINK format files were created using Illumina GenomeStudio v2.0.4.

During the quality control, individuals with a call-rate of < 95% or mismatching sex, defined based on the heterozygosity of the X chromosome and sex in phenotype data, were excluded from the analysis. Variants were filtered by call-rate < 95% and HWE p-value < 10^-4^ (autosomal variants only). Variant positions were updated to the Genome Reference Consortium Human Build 37 and all variants were changed to be from TOP strand using reference information provided by Dr. Will Rayner from the University of Oxford (https://www.well.ox.ac.uk/~wrayner/strand/).

Variants with MAF<1% and Indels were removed before imputation. Prephasing was carried out using the Eagle v2.3 software ^73^(number of conditioning haplotypes Eagle2 uses when phasing each sample was set to: --Kpbwt=20000) and imputation was executed using Beagle v.28Sep18.793 ^74,75^with an effective population size ne=20,000. As a reference, the Estonian population specific imputation reference of 2297 WGS samples was used ^76^.

*Phenotype definitions*

The smoking phenotype included in the genome-wide association (GWAS) analysis was defined based on the EstBB Mental Health online Survey (MHoS) self-reported data, collected in spring 2021.

Cases of smoking were defined if they had reported to have ever regularly used tobacco products (cigarettes, oral tobacco, electronic cigarettes, pipe tobacco, nicotine pouches) either monthly, weekly, every day, or almost every day. We identified 36,589 cases with a lifetime history of regular use of tobacco products (n=13,848 men, n=22,741 women) and 44,466 controls (n= 9820 men, n=34,646 women) who reported not having any history of regular use of tobacco products.

*Genome-wide association analysis*

We conducted the GWASs using the REGENIE software (version  2.2.4) ^77^ adjusting for the first ten principal components of the genotype matrix, as well as for birth year, and sex.

##

## **References**

59 McCarthy S, Das S, Kretzschmar W, Delaneau O, Wood AR, Teumer A *et al.* A reference panel of 64,976 haplotypes for genotype imputation. *Nat Genet* 2016; **48**: 1279–1283.

60 UK10K Consortium, Walter K, Min JL, Huang J, Crooks L, Memari Y *et al.* The UK10K project identifies rare variants in health and disease. *Nature* 2015; **526**: 82–90.

61 Millard LAC, Davies NM, Gaunt TR, Davey Smith G, Tilling K. Software Application Profile: PHESANT: a tool for performing automated phenome scans in UK Biobank. *Int J Epidemiol* 2018; **47**: 29–35.

62 Boomsma DI, Willemsen G, Sullivan PF, Heutink P, Meijer P, Sondervan D *et al.* Genome-wide association of major depression: description of samples for the GAIN Major Depressive Disorder Study: NTR and NESDA biobank projects. *Eur J Hum Genet* 2008; **16**: 335–342.

63 Mbarek H, Milaneschi Y, Hottenga J-J, Ligthart L, de Geus EJC, Ehli EA *et al.* Genome-Wide Significance for PCLO as a Gene for Major Depressive Disorder. *Twin Res Hum Genet* 2017; **20**: 267–270.

64 Fedko IO, Hottenga J-J, Medina-Gomez C, Pappa I, van Beijsterveldt CEM, Ehli EA *et al.* Estimation of Genetic Relationships Between Individuals Across Cohorts and Platforms: Application to Childhood Height. *Behav Genet* 2015; **45**: 514–528.

65 Boomsma DI, Wijmenga C, Slagboom EP, Swertz MA, Karssen LC, Abdellaoui A *et al.* The Genome of the Netherlands: design, and project goals. *Eur J Hum Genet* 2014; **22**: 221–227.

66 Deelen P, Menelaou A, van Leeuwen EM, Kanterakis A, van Dijk F, Medina-Gomez C *et al.* Improved imputation quality of low-frequency and rare variants in European samples using the ‘Genome of The Netherlands’. *Eur J Hum Genet* 2014; **22**: 1321–1326.

67 Price AL, Patterson NJ, Plenge RM, Weinblatt ME, Shadick NA, Reich D. Principal components analysis corrects for stratification in genome-wide association studies. *Nat Genet* 2006; **38**: 904–909.

68 Abdellaoui A, Hottenga J-J, de Knijff P, Nivard MG, Xiao X, Scheet P *et al.* Population structure, migration, and diversifying selection in the Netherlands. *Eur J Hum Genet* 2013; **21**: 1277–1285.

69 Das S, Forer L, Schönherr S, Sidore C, Locke AE, Kwong A *et al.* Next-generation genotype imputation service and methods. *Nat Genet* 2016; **48**: 1284–1287.

70 Yang J, Lee SH, Goddard ME, Visscher PM. GCTA: a tool for genome-wide complex trait analysis. *Am J Hum Genet* 2011; **88**: 76–82.

71 Schumann G, Liu C, O’Reilly P, Gao H, Song P, Xu B *et al.* KLB is associated with alcohol drinking, and its gene product β-Klotho is necessary for FGF21 regulation of alcohol preference. *Proc Natl Acad Sci U S A* 2016; **113**: 14372–14377.

72 Evangelou E, Gao H, Chu C, Ntritsos G, Blakeley P, Butts AR *et al.* New alcohol-related genes suggest shared genetic mechanisms with neuropsychiatric disorders. *Nat Hum Behav* 2019; **3**: 950–961.

73 Loh P-R, Danecek P, Palamara PF, Fuchsberger C, A Reshef Y, K Finucane H *et al.* Reference-based phasing using the Haplotype Reference Consortium panel. *Nat Genet* 2016; **48**: 1443–1448.

74 Browning SR, Browning BL. Rapid and accurate haplotype phasing and missing-data inference for whole-genome association studies by use of localized haplotype clustering. *Am J Hum Genet* 2007; **81**: 1084–1097.

75 Browning BL, Zhou Y, Browning SR. A One-Penny Imputed Genome from Next-Generation Reference Panels. *Am J Hum Genet* 2018; **103**: 338–348.

76 Mitt M, Kals M, Pärn K, Gabriel SB, Lander ES, Palotie A *et al.* Improved imputation accuracy of rare and low-frequency variants using population-specific high-coverage WGS-based imputation reference panel. *Eur J Hum Genet* 2017; **25**: 869–876.

77 Mbatchou J, Barnard L, Backman J, Marcketta A, Kosmicki JA, Ziyatdinov A *et al.* Computationally efficient whole-genome regression for quantitative and binary traits. *Nat Genet* 2021; **53**: 1097–1103.
