# Supplementary material for: Neandertal introgression partitions the genetic landscape of neuropsychiatric disorders and associated behavioral phenotypes
Source: Transl Psychiatry. 2022 Oct 5;12:433. doi: 10.1038/s41398-022-02196-2 (PMC9534885; doi:10.1038/s41398-022-02196-2)
Supplement: Supplementary file 2 — Supplementary Tables Legends [file 41398_2022_2196_MOESM2_ESM.docx]

## **Supplementary Tables LEGENDS**

**Table S1: Neandertal DNA association enrichment results**

For each tested phenotype and for various significance association P value cutoffs the enrichment results in the form of average ORs (with 95% CIs and number of tag aSNPs with association P value below the significance cutoff) and empirical enrichment P values are displayed.

**Table S2: Neandertal marker SNPs**

Text file with genotype information for aSNPs that were included in this study.

**Table S3: Genome-wide significant Neandertal DNA risk loci**

Neandertal DNA associations with P<5x10^-8^ among the sets of tested phenotypes in this study, together with their associated GWAS summary statistics, inferred Neandertal haplotype, and overlapping eQTL and missense variants are provided.

**Table S4: Classification of medication GWAS in the UK Biobank**

Medication GWAS from the UK Biobank are annotated based on three levels of WHO classifiers. This table contains only medication that was assigned to WHO nervous disease meta-classifier ‘N’.

**Table S5: Proportional Neandertal DNA association for groups of behavioral phenotypes**

The percentage of average odds ratio (OR) greater than one in eight groups of UK Biobank phenotype, the group of smoking phenotypes in the Biobank Japan and the group of tested NESDA phenotypes are provided. For each proportion of average ORs>1 a binomial CI together with its P value (tested against hypothesis of 50%) and false discovery rate (FDR) is available.
